# TRIM28 negatively regulates the RLR signaling pathway by targeting MAVS for degradation *via* K48-linked polyubiquitination

**DOI:** 10.1016/j.jbc.2023.104660

**Published:** 2023-04-27

**Authors:** Ya-Yun Chen, Xiang-Hong Ran, Run-Ze Ni, Dan Mu

**Affiliations:** Institute of Life Sciences, Chongqing Medical University, Chongqing, China

**Keywords:** TRIM28, MAVS, degradation, ubiquitination, RLR signaling

## Abstract

Mitochondrial antiviral signaling (MAVS) protein is a core signaling adapter in the retinoid acid–inducible gene-I–like receptor (RLR) signaling pathway that recruits downstream signaling factors, ultimately leading to the activation of type Ⅰ interferons. However, the mechanisms that modulate the RLR signaling pathway by manipulating MAVS are not fully understood. Previous studies suggested that tripartite motif 28 (TRIM28) participates in regulating innate immune signaling pathways by inhibiting the expression of immune-related genes at the transcriptional level. In this study, we characterized TRIM28 as a negative regulator of the RLR signaling pathway in a MAVS-dependent manner. Overexpression of TRIM28 inhibited the MAVS-induced production of type Ⅰ interferons and proinflammatory cytokines, while knocking down TRIM28 exerted the opposite effect. Mechanistically, TRIM28 targeted MAVS for proteasome-mediated degradation *via* K48-linked polyubiquitination. The RING domain of TRIM28, especially the cysteine residues at positions 65 and 68, was critical for the suppressive effect of TRIM28 on MAVS-mediated RLR signaling, while each of the C-terminal domains of TRIM28 contributed to its interaction with MAVS. Further investigation revealed that TRIM28 transferred ubiquitin chains to the K7, K10, K371, K420, and K500 residues of MAVS. Together, our results reveal a previously uncharacterized mechanism involving TRIM28 in fine-tuning innate immune responses and provide new insights into the mechanisms by which MAVS is regulated, which contribute to the understanding of the molecular mechanisms underlying immune homeostasis maintenance.

Innate immunity is the first barrier of the body against infection by pathogenic microorganisms, which cooperates with adaptive immunity to prevent foreign invasions. When invasion occurs, innate immunity is activated, and pathogen-associated molecular patterns are recognized by cellular pattern recognition receptors (1, 2), which initiate innate immune signaling and ultimately promote the immune response. Among all kinds of pattern recognition receptors, retinoid acid–inducible gene (RIG)-I–like receptors (RLRs), including RIG-I and melanoma differentiation-associated gene 5, function as cytoplasmic viral RNA sensors to recognize viral RNA and interact with mitochondrial antiviral signaling (MAVS, also known as IPS1, VISA, or CARDIF) protein, which subsequently stimulates downstream signaling events (3, 4, 5). MAVS then activates two cytosolic protein kinase complexes, including TBK1 (TANK-binding kinase 1) or inhibitor of nuclear factor κB kinase ε and the IKK complex, which leads to the activation of interferon regulatory factor 3 (IRF3) or IRF7 and nuclear factor-κB (NF-κB), ultimately inducing the expression of type Ⅰ interferons (IFNs) and the production of proinflammatory cytokines (6, 7). During DNA viral infection, cyclic GMP-AMP synthase recognizes cytoplasmic double-stranded DNA and activates stimulator of interferon genes (STING), which also leads to the activation of TBK1/inhibitor of nuclear factor κB kinase ε and the ultimate activation of IFNs (1). To prevent excessive host innate immune responses, NF-κB activation and type Ⅰ IFN signaling must be tightly regulated (8). However, the molecular mechanisms underlying immune homeostasis are not fully understood.

Ubiquitination, a posttranslational protein modification, is one of the most important regulatory mechanisms in the positive or negative regulation of innate immune signaling pathways (9). Various types of ubiquitination are involved in signal transduction. For example, RIG-I can be activated through K63-linked ubiquitination by cyclophilin A and a RING-type E3 ubiquitin ligase tripartite motif containing 25 (TRIM25) (10, 11). MAVS has been reported to be targeted by a HECT domain-containing E3 ligase AIP4, a RING-type E3 ubiquitin ligase RNF125 and TRIM25 for degradation *via* K48-linked polyubiquitination (12, 13, 14).

TRIM28 (also known as KAP1 or TIF1β), which is a member of a large family of E3 ubiquitin ligases consisting of more than 80 proteins, has been reported to be involved in various cellular processes, such as transcriptional regulation, DNA damage repair, and maintenance of the heterochromatin environment (15). During the past decade, increasing evidence has shown that TRIM28 participates in regulating innate immune signaling pathways (16, 17, 18, 19). TRIM28 was demonstrated to inhibit the induction of type Ⅰ IFNs by targeting IRF7 for SUMOylation after viral infections (16), suppress interleukin (IL)-6–induced STAT3-dependent gene expression by dephosphorylating STAT3 (17), or deacetylate NF-κB/p65 by disrupting the interactions among STAT3, p300, and NF-κB/p65, leading to reduced IL-6 production (18). Among all of these studies, TRIM28 was characterized as a negative regulator of the expression of immune-related genes at the transcriptional level. However, a recent study has provided a new clue that TRIM28 may employ other mechanisms to repress innate immune responses by showing that TRIM28 abolishes the suppressive effect on the IFN-β promoter in MAVS-KO cells (19). However, the detailed molecular mechanisms by which TRIM28 mediates MAVS-dependent IFN responses remain elusive.

In the present study, we demonstrate that TRIM28 targets MAVS for degradation *via* the ubiquitin–proteasome pathway, thus negatively regulating RIG-I/MAVS signaling. TRIM28 strongly hampered Sendai virus (SeV, an RNA virus)–induced production of type Ⅰ IFNs and proinflammatory cytokines in a MAVS-dependent manner. Mechanistically, TRIM28 directly interacted and colocalized with MAVS and facilitated the degradation of MAVS but not that of RIG-I or downstream factors *via* K48-linked polyubiquitination. Further investigation revealed that the RING domain of TRIM28, especially the cysteine residues at positions 65 and 68, was critical for transferring K48-linked polyubiquitin chains to several key lysine residues of MAVS. On the other hand, the C-terminal domains of TRIM28 mediated its interaction with MAVS. Thus, these findings led to the identification of the E3 ubiquitin ligase TRIM28 as a negative regulator of MAVS-dependent IFN responses by promoting MAVS degradation *via* K48-linked ubiquitination, providing new insights into the role of TRIM28 in maintaining the cellular homeostasis of innate immunity.

## Results

### TRIM28 negatively regulates RIG-I‒MAVS-mediated signaling

**Figure 1:**
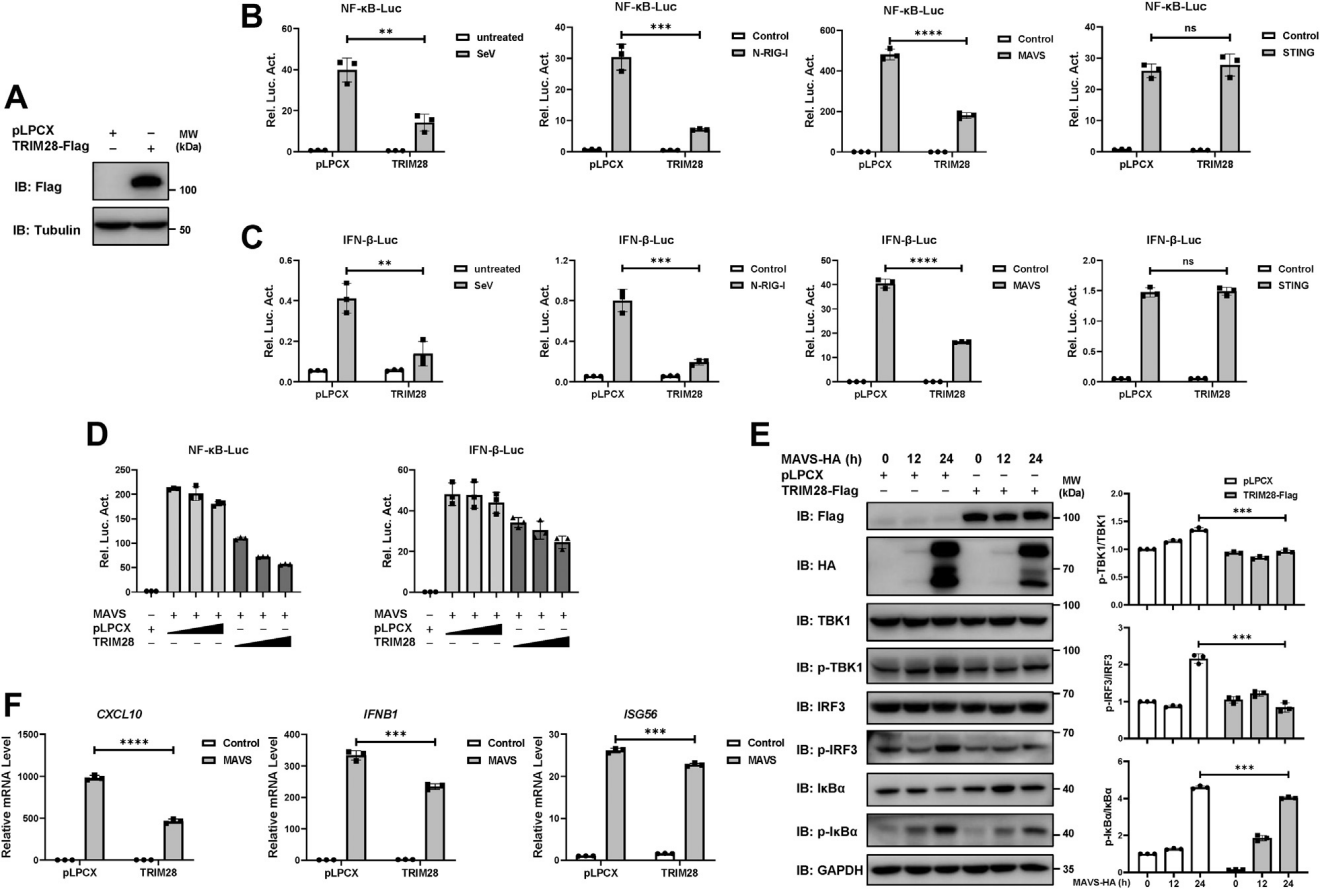
**Overexpression of TRIM28 inhibits the activation of RLR signaling pathway through the RIG-I‒MAVS axis.***A*, Western blot of HEK293T cells transfected with Flag-tagged TRIM28 or empty vector pLPCX. *B* and *C*, plasmids encoding Flag-tagged TRIM28 or an empty vector was transfected into 293T cells with NF-κB-luc (*B*) or IFN-β-luc (*C*) reporter constructs. Cells were treated with Sendai Virus (SeV) (multiplicity of infection [MOI] = 1) for 24 h or cotransfected with a plasmid expressing N-RIG-I, STING, or MAVS as stimuli. A pRL-TK *Renilla* luciferase reporter was transfected along to normalize transfection efficiency. *D*, increasing amounts of TRIM28 or empty vector pLPCX were cotransfected into 293T cells with MAVS, together with NF-κB-luc or IFN-β-luc constructs. Forty-eight hours posttransfection, the cells were collected, and luciferase activity was measured by a dual‒luciferase assay. *E*, 293T cells were transfected with Flag-tagged TRIM28 or an empty vector, together with HA-tagged MAVS at the indicated times. Western blot was performed to detect the protein expression and phosphorylation of TBK1, IRF3, and IκBα with the indicated antibodies (*left*). Ratio of phosphorylated protein to total protein level was calculated and normalized to control (*right*). *F*, RT‒qPCR analyses of *CXCL10*, *IFNB1*, and *ISG56* mRNA expression levels in 293T cells transfected with MAVS and TRIM28 or an empty vector. Data from three independent experiments were shown as mean ± SD and analyzed by two-sided unpaired *t* test. ∗∗*p* < 0.01; ∗∗∗*p* < 0.001; ∗∗∗∗*p* < 0.0001; and ns, not significant. HA, hemagglutinin; IFN, interferon; IRF3, interferon regulatory factor 3; MAVS, mitochondrial antiviral signaling; RIG, retinoid acid–inducible gene; RLR, RIG-I–like receptor; STING, stimulator of interferon genes; TBK1, TANK-binding kinase 1; TRIM28, tripartite motif 28.

We first determined the effect of TRIM28 on SeV-induced RLR signaling with dual luciferase reporter assays. NF-κB- or IFN-β-responsive luciferase (luc) reporter constructs were cotransfected with a plasmid expressing Flag-tagged TRIM28. The expression of the Flag-tagged TRIM28 construct was verified by Western blot (Fig. 1*A*). The reporter assays showed that overexpression of TRIM28 inhibited the activation of the NF-κB-luc and IFN-β-luc reporters induced by SeV (Fig. 1, *B* and *C*). Next, we coexpressed TRIM28 with various signaling transducers, including N-RIG-I (a constitutively active N-terminal portion of RIG-I), MAVS, or STING, to determine at which signaling axis TRIM28 takes effect. The results showed that TRIM28 overexpression hampered the activation of the NF-κB-luc and IFN-β-luc reporters induced by N-RIG-I and MAVS but not by STING (Fig. 1, *B* and *C*), indicating that TRIM28 is exclusively involved in the RNA-sensing signaling pathway through the RIG-I‒MAVS axis. We further showed that TRIM28 downregulated MAVS-mediated activation of NF-κB-luc and IFN-β-luc reporters in a dose-dependent manner (Fig. 1*D*). We then examined the effect of TRIM28 on MAVS-mediated activation of the RLR signaling pathway by analyzing the phosphorylation levels of downstream factors at the indicated times. As expected, overexpression of MAVS enhanced the phosphorylation levels of TBK1, IRF3, and IκBα (Fig. 1*E*). However, in the presence of TRIM28, the MAVS-induced enhancement of TBK1, IRF3, and IκBα phosphorylation was attenuated (Fig. 1*E*). The effect of TRIM28 on MAVS-induced proinflammatory cytokines and the production of type Ⅰ IFNs and IFN-stimulated genes (ISGs) were also assessed. RT‒qPCR analyses showed that overexpression of TRIM28 significantly reduced the MAVS-induced mRNA expression levels of *CXCL10*, *IFNB1*, and *ISG56* (Fig. 1*F*).

**Figure 2:**
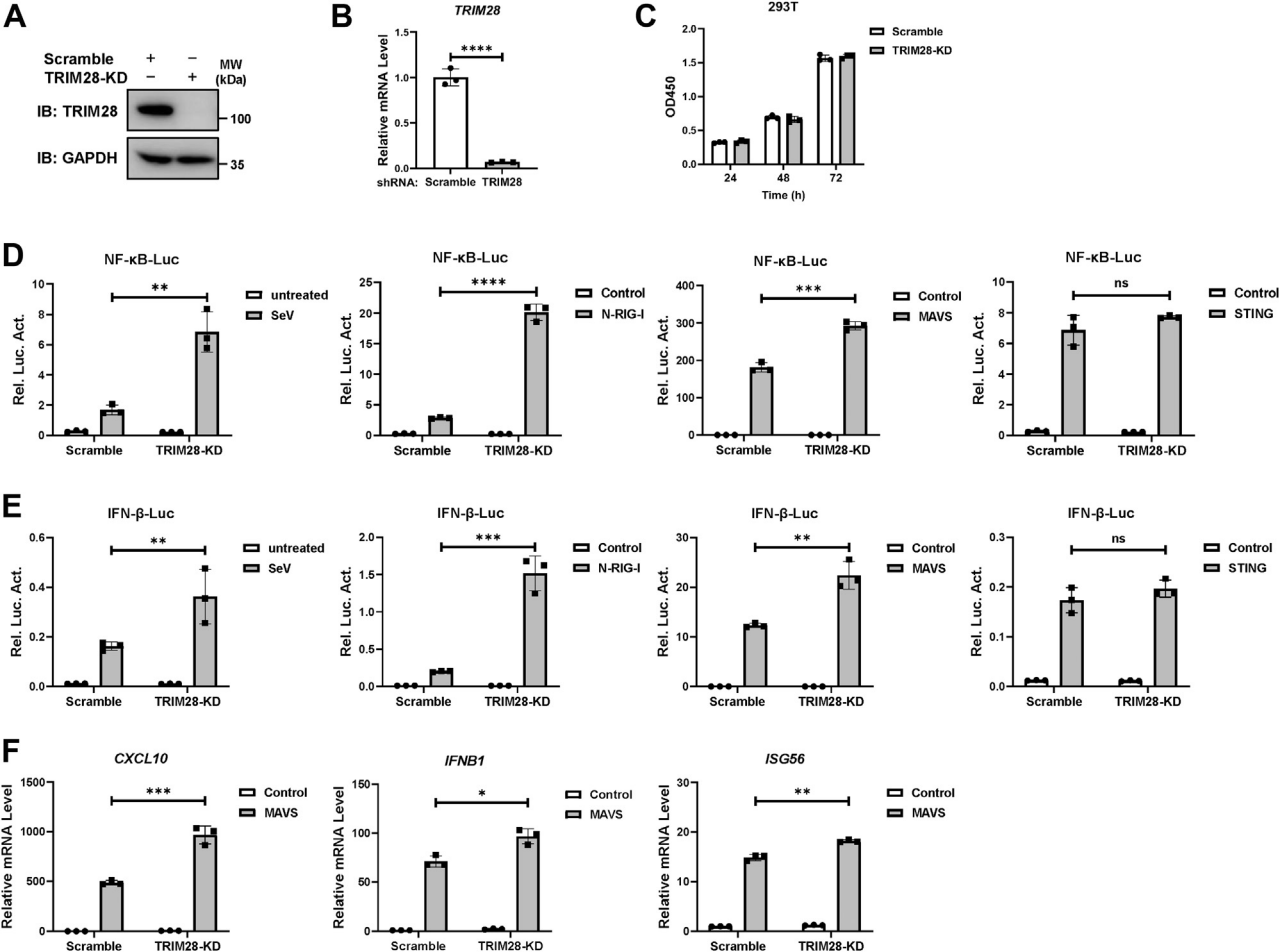
**TRIM28 depletion potentiates MAVS-mediated RLR signaling responses.***A*‒*C*, HEK293T cells were stably transduced with shRNA targeting TRIM28 (TRIM28-KD). Scrambled shRNA was used as a control. The knockdown efficacy of endogenous TRIM28 was validated by Western blot (*A*) and qPCR assays (*B*), with GAPDH as a loading control. The cell viability was detected by CCK-8 assay (*C*). *D* and *E*, NF-κB-luc (*D*) or IFN-β-luc (*E*) reporter was transfected into TRIM28-KD or control scramble-KD 293T cells. Cells were treated with SeV (MOI = 1) for 24 h or cotransfected with a plasmid expressing N-RIG-I, STING, or MAVS as stimuli. Luciferase activity was determined at 24 h poststimulation using the dual luciferase reporting assay. *F*, RT‒qPCR analyses of *CXCL10*, *IFNB1*, and *ISG56* mRNA expression levels in TRIM28-KD or scramble-KD 293T cells transfected with a plasmid encoding MAVS or an empty vector. All data are representative of three independent experiments and are presented as the mean ± SD (n = 3). The statistical significance analyses were performed using two-sided unpaired *t* test. ∗*p* < 0.05; ∗∗*p* < 0.01; ∗∗∗*p* < 0.001; ∗∗∗∗*p* < 0.0001; and ns, not significant. IFN, interferon; MAVS, mitochondrial antiviral signaling; MOI, multiplicity of infection; RIG, retinoid acid–inducible gene; RLR, RIG-I–like receptor; SeV, Sendai virus; shRNA, short hairpin RNA; STING, stimulator of interferon genes; TRIM28, tripartite motif 28.

To confirm the results above, we examined the effect of TRIM28 depletion on MAVS-mediated RLR signaling by employing a lentiviral-specific short hairpin RNA (shRNA) targeting TRIM28. The efficacy of TRIM28 knockdown in HEK293T cells was validated by Western blot and qPCR assays (Fig. 2, *A* and *B*). The CCK-8 assays showed that knockdown of TRIM28 exerted no significant influence on cell viability (Fig. 2*C*). Consistent with the results from the overexpression experiments, TRIM28 depletion greatly enhanced the activation of NF-κB-luc and IFN-β-luc reporters mediated by SeV, N-RIG-I, and MAVS but not by STING (Fig. 2, *D* and *E*). Moreover, TRIM28 knockdown augmented the MAVS-induced expression of *CXCL10*, *IFNB1*, and *ISG56* (Fig. 2*F*). Taken together, these data indicate that TRIM28 exerts a strong suppressive effect on RIG-I‒MAVS–mediated signaling.

### The suppressive effect of TRIM28 on RLR signaling is dependent on MAVS

**Figure 3:**
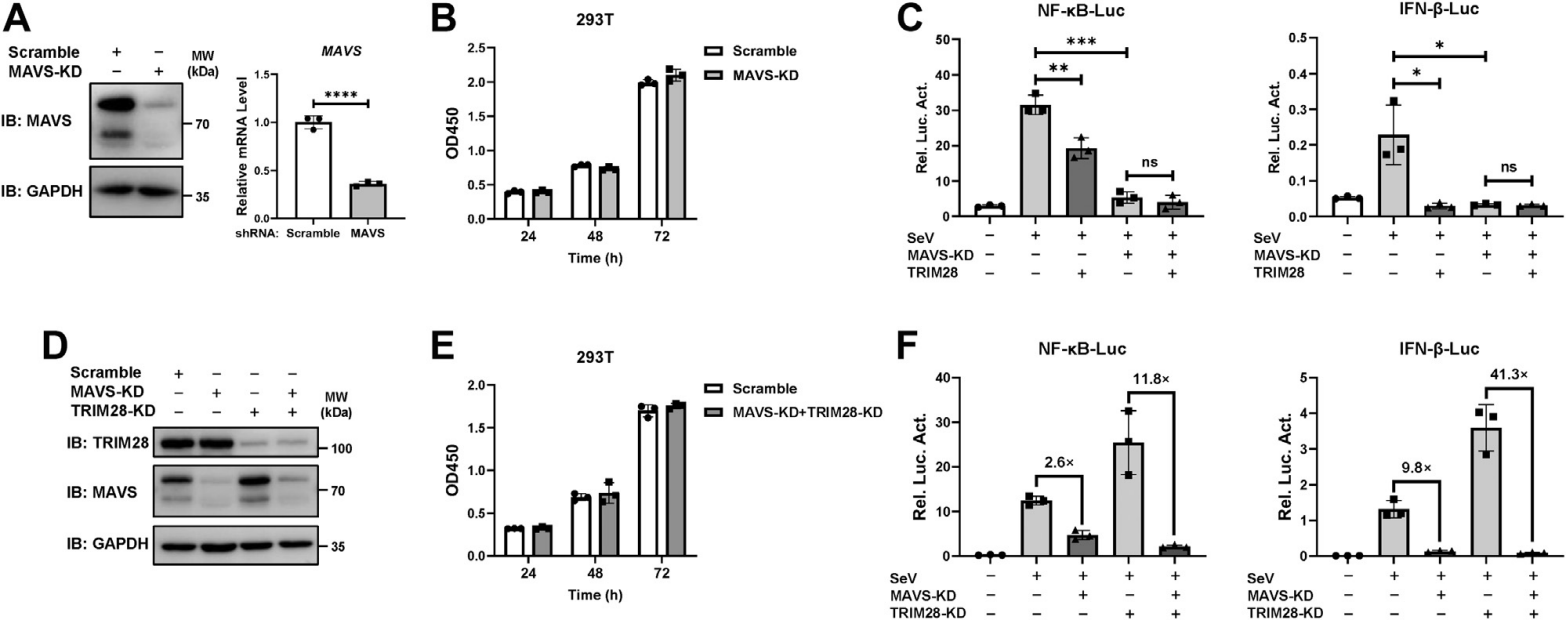
**Suppressive effect of TRIM28 on RLR signaling is dependent on MAVS.***A*, the knockdown effect of endogenous MAVS in HEK293T cells was confirmed by Western blot and qPCR assays. GAPDH served as a loading control. *B*, the cell viability of MAVS-KD or scramble-KD 293T cells was measured by using CCK-8 assays. *C*, MAVS-KD or scramble-KD 293T cells were seeded in 96-well plates and cotransfected with TRIM28 or a pLPCX vector, together with the NF-κB-luc or IFN-β-luc construct and the pRL-TK plasmid. Cells were then infected with SeV for 24 h before luciferase analysis. *D* and *E*, double knockdown of MAVS and TRIM28 (MAVS-KD+TRIM28-KD) 293T cells were generated. The knockdown efficiencies of the indicated proteins were confirmed by Western blot (*D*). The cell viability of MAVS and TRIM28 double KD or scramble-KD 293T cells was measured by using CCK-8 assays (*E*). *F*, the knockdown 293T cells were cotransfected with the NF-κB-luc or IFN-β-luc construct and the pRL-TK plasmid and were then infected with SeV (MOI = 1) at 24 h posttransfection. Cells were cultured for another 24 h and dual‒luciferase reporter assays were performed. Data are presented as the mean ± SD, representative of experiments performed in triplicate. The significance was analyzed by two-sided unpaired *t* test. ∗*p* < 0.05; ∗∗*p* < 0.01; ∗∗∗*p* < 0.001; ∗∗∗∗*p* < 0.0001; and ns, not significant. IFN, interferon; MAVS, mitochondrial antiviral signaling; NF-κB, nuclear factor-κB; RIG, retinoid acid–inducible gene; RLR, RIG-I–like receptor; SeV, Sendai virus; TRIM28, tripartite motif 28.

To investigate whether the negative regulation of RLR signaling by TRIM28 is dependent on MAVS, we examined the effect of TRIM28 on SeV-induced NF-κB-luc and IFN-β-luc reporter activities under MAVS depletion conditions. A lentiviral shRNA delivery system was employed to generate MAVS-knockdown 293T cells. The protein and mRNA expression levels of MAVS in cells transduced with MAVS-targeted shRNA were greatly decreased compared to that in cells transduced with scramble shRNA (Fig. 3*A*). MAVS knockdown showed no impairment of cell viability (Fig. 3*B*). As previously reported (7), depletion of MAVS remarkably attenuated the activation of the NF-κB-luc and IFN-β-luc reporters induced by SeV (Fig. 3*C*). Notably, knockdown of MAVS abrogated the inhibitory effect of TRIM28 on SeV-induced NF-κB and IFN-β signaling (Fig. 3*C*), suggesting that TRIM28 negatively regulates SeV-induced RLR signaling in a MAVS-dependent manner. We also simultaneously knocked down MAVS and TRIM28 in 293T cells to further determine the effect of TRIM28 on MAVS-mediated RLR signaling. Western blot was performed to verify the reduction in the indicated protein levels (Fig. 3*D*). CCK-8 assays showed that knocking down both MAVS and TRIM28 had little effect on cell viability (Fig. 3*E*). Dual‒luciferase reporter assays showed that knocking down MAVS caused a 2.6-fold decrease in SeV-induced NF-κB-luc reporter activity and a 9.8-fold decrease in SeV-induced IFN-β-luc reporter activity, while MAVS/TRIM28 double knockdown led to a 11.8- and a 41.3-fold decrease in SeV-activated NF-κB-luc and IFN-β-luc reporter activity, respectively (Fig. 3*F*). These data agree with the results shown in Figure 2 indicating that the knockdown of TRIM28 potentiates the MAVS-mediated activation of RLR signaling.

### TRIM28 targets MAVS for proteasome-mediated degradation

**Figure 4:**
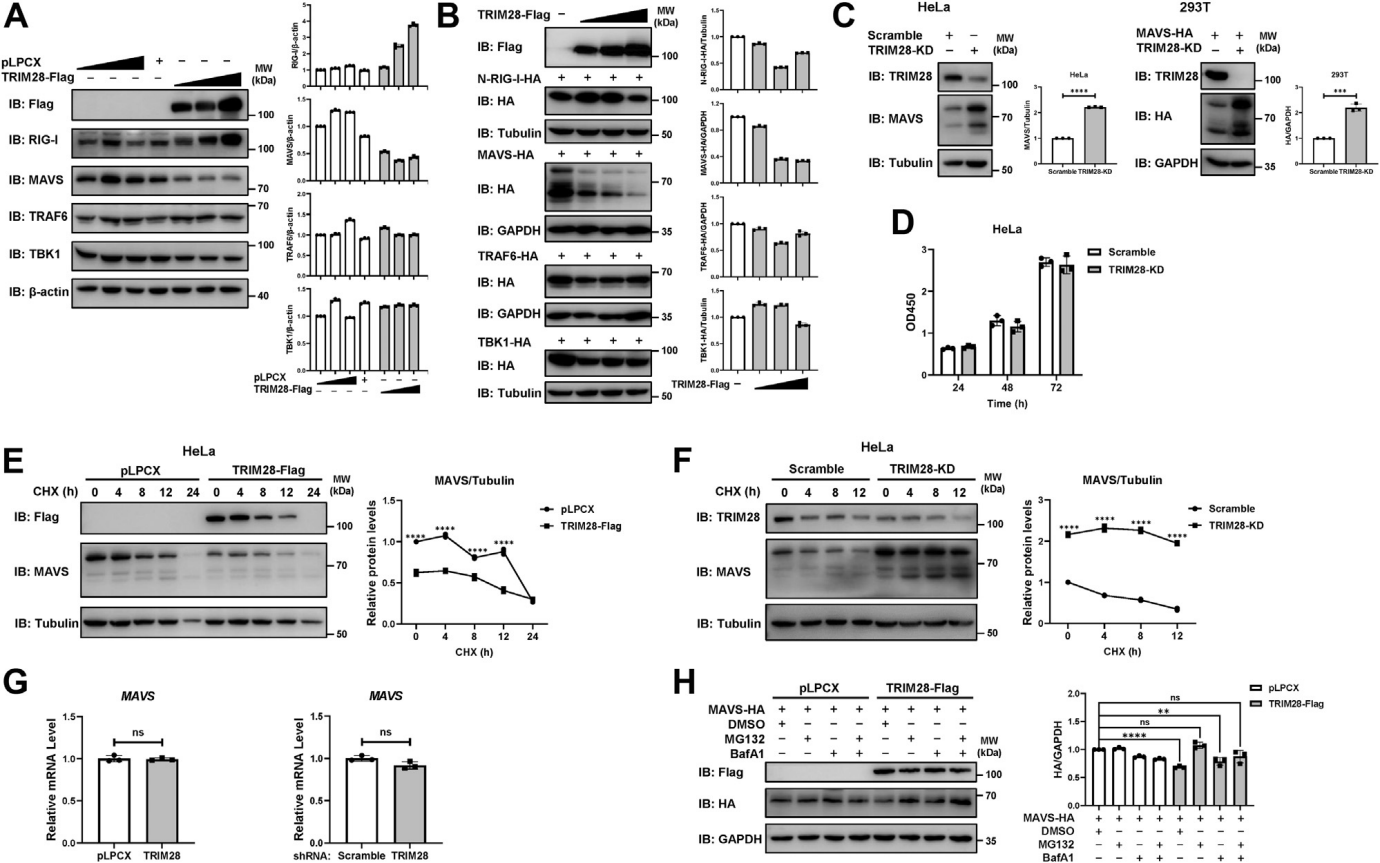
**TRIM28 targets MAVS for proteasome-mediated degradation.***A*, immunoblot analysis of HEK293T cells transfected with increasing doses of Flag-tagged TRIM28 or an empty vector. Western blot was performed to detect the protein levels of key factors with the indicated antibodies. *B*, increasing amounts of plasmid encoding Flag-tagged TRIM28 and equal amounts of plasmids expressing HA-tagged N-RIG-I, MAVS, TRAF6 or TBK1 were cotransfected into 293T cells. The expression levels of exogenous proteins were immunodetected using an anti-HA antibody. The relative protein levels were normalized to those of the indicated internal reference. *C*, the effect of TRIM28 knockdown on the protein expression levels of endogenous MAVS in TRIM28-KD HeLa cells or exogenous MAVS in TRIM28-KD 293T cells. Immunoblot analyses were performed with an anti-MAVS or an anti-HA antibody, respectively. *D*, CCK-8 assays were performed to measure the cell viability of TRIM28-KD or scramble-KD HeLa cells. *E*, CHX chase analyses of endogenous MAVS in HeLa cells transfected with Flag-tagged TRIM28 or pLPCX. Cells were treated with 100 μg/ml CHX for the time points indicated. The immunoblot values of MAVS expression were quantified, normalized to Tubulin, and then normalized to the control. *F*, CHX chase analyses of endogenous MAVS in TRIM28-KD or scramble-KD HeLa cells. Cells were treated with 100 μg/ml CHX for the indicated durations. The relative expression levels of MAVS were quantified. *G*, comparison of *MAVS* mRNA levels in TRIM28-overexpressing or TRIM28-KD 293T cells with those in control cells. *H*, immunoblot analysis of lysates in 293T cells transfected with MAVS-HA and TRIM28-Flag or empty vector and then treated with MG132 (10 μM), bafilomycin A1 (BafA1; 5 nM) or DMSO, or cotreated with MG132 and BafA1 for 6 h. The immunoblots were quantified, normalized to GAPDH levels. Data from three independent experiments were presented as mean ± SD and statistical significance was calculated by two-sided unpaired *t* test. ∗∗*p* < 0.01; ∗∗∗*p* < 0.001; ∗∗∗∗*p* < 0.0001; and ns, not significance. CHX, cycloheximide; DMSO, dimethyl sulfoxide; HA, hemagglutinin; MAVS, mitochondrial antiviral signaling; RIG, retinoid acid–inducible gene; TBK1, TANK-binding kinase 1; TRAF6, tumor necrosis factor receptor–associated factor 6; TRIM28, tripartite motif 28.

We next explored the specific mechanisms by which TRIM28 regulates MAVS to inhibit RLR signaling. HEK293T cells were transfected with increasing amounts of Flag-tagged TRIM28, and the endogenous expression levels of key modulators involved in RLR signaling were examined. The results showed that overexpression of TRIM28 led to a significant decrease in the endogenous protein levels of MAVS, but not in those of RIG-I or downstream factors, such as tumor necrosis factor receptor–associated factor 6 (TRAF6) or TBK1 (Fig. 4*A*). We then confirmed this finding by ectopically coexpressing TRIM28 with hemagglutinin (HA)-tagged MAVS, N-RIG-I, TRAF6, or TBK1. A dose-dependent reduction in the protein levels of exogenous MAVS but not those of other factors was observed (Fig. 4*B*). Consistently, knockdown of TRIM28 in HeLa cells led to a dramatic increase in MAVS protein expression (Fig. 4*C*). The CCK-8 assays indicated that TRIM28 knockdown in HeLa cells exerted no effect on cell viability (Fig. 4*D*). The translation inhibitor cycloheximide (CHX), which blocks protein synthesis, was used to evaluate the influence of TRIM28 on the protein stability of MAVS in HeLa cells. The CHX-chase assay results showed that TRIM28 overexpression accelerated the degradation of MAVS (Fig. 4*E*), while TRIM28 depletion enhanced MAVS protein stability (Fig. 4*F*). In addition, we evaluated the effect of TRIM28 on the mRNA levels of MAVS and showed that MAVS mRNA levels were not affected by TRIM28 (Fig. 4*G*). Together, these observations indicate that TRIM28 negatively regulates the expression of MAVS at the protein level.

The ubiquitin‒proteasome system is one of the most crucial mechanisms involved in protein degradation in eukaryotic cells, and E3 ligases are considered to be indispensable in this process due to their specific substrate binding (20). TRIM28 has been identified as an E3 ubiquitin ligase owing to its RING domain and has been reported to be involved in the regulation of ubiquitination (21). TRIM28 has also been reported to activate autophagy (22), which leads to protein degradation (23). To explore whether TRIM28 promotes MAVS degradation *via* the ubiquitin‒proteasome pathway or the autophagy‒lysosome pathway, 293T cells were transfected with HA-tagged MAVS and Flag-tagged TRIM28 or empty vector and then treated with the proteasome inhibitor MG132, autolysosome inhibitor bafilomycin A1 (BafA1), or cotreated with MG132 and BafA1. As shown in Figure 4*H*, the degradation of MAVS induced by TRIM28 overexpression was blocked by MG132 treatment as well as by cotreatment with MG132 and BafA1, but not BafA1 treatment, suggesting that TRIM28 mediated MAVS degradation *via* the ubiquitin–proteasome pathway.

### TRIM28 facilitates the K48-linked polyubiquitination of MAVS

**Figure 5:**
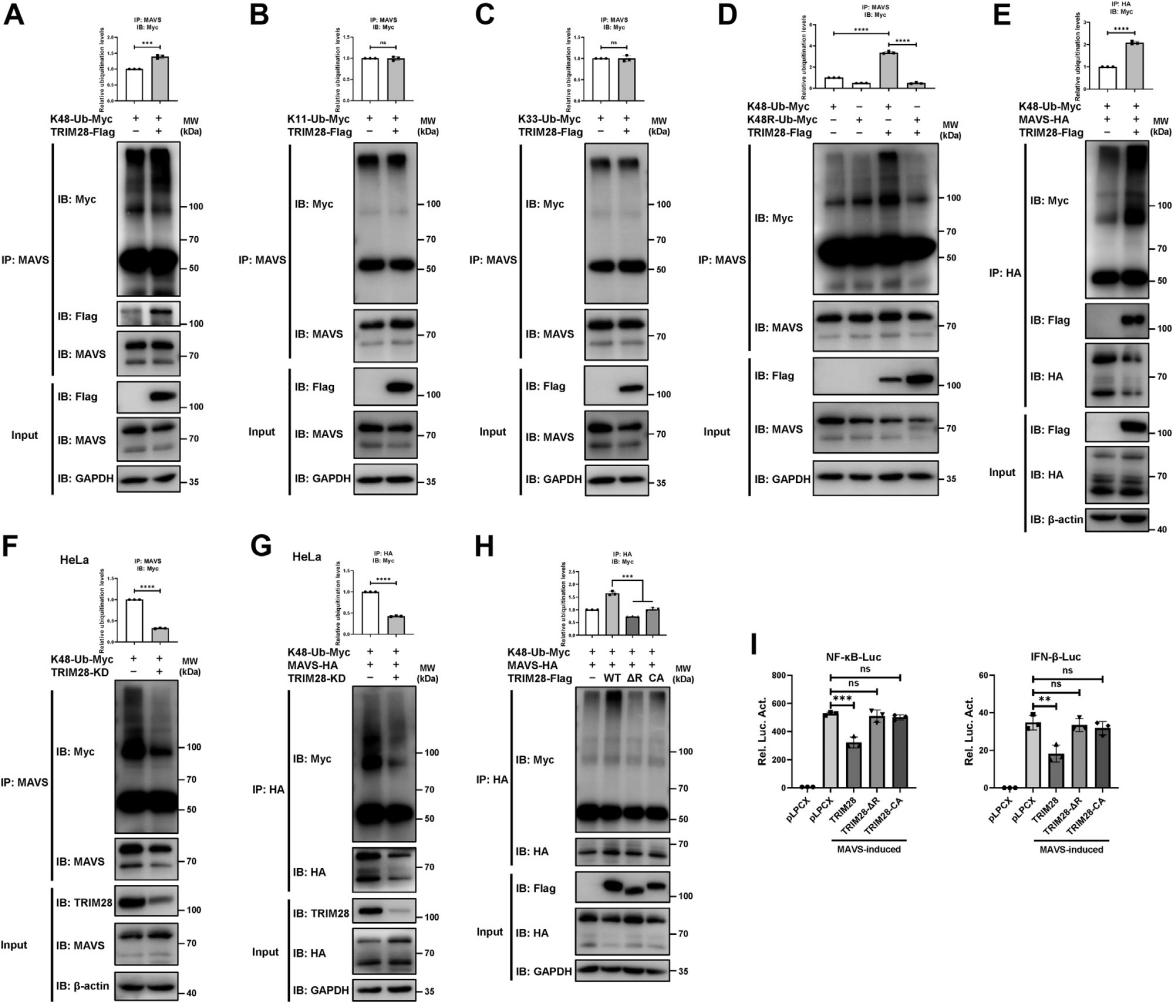
**TRIM28 facilitates the K48-linked polyubiquitination of MAVS.***A*‒*D*, immunoblot analysis of HEK293T cells transfected with K48-ubiquitin (Ub)-Myc (*A*), K11-Ub-Myc (*B*), K33-Ub-Myc (*C*) or K48R-Ub-Myc (*D*), and Flag-tagged TRIM28 or pLPCX. The cell lysates were immunoprecipitated with an anti-MAVS antibody and the levels of ubiquitination were measured with an anti-Myc antibody. *E*, 293T cells were cotransfected with K48-Ub-Myc and HA-tagged MAVS, together with Flag-tagged TRIM28 or pLPCX. MAVS-HA protein were immunoprecipitated using an anti-HA antibody and its ubiquitination was determined with an anti-Myc antibody. *F* and *G*, TRIM28-KD or scramble-KD HeLa cells were transfected with K48-Ub-Myc (*F*) or K48-Ub-Myc and MAVS-HA (*G*). Ubiquitination levels were detected by Co-IP with an anti-MAVS antibody (*F*) or an anti-HA antibody (*G*) and immunoblotted with an anti-Myc antibody. *H*, plasmids encoding Flag-tagged WT or mutant TRIM28 were cotransfected with K48-Ub-Myc and MAVS-HA into 293T cells. Ubiquitination levels were measured by Western blot with an anti-Myc antibody. The relative ubiquitination levels were normalized to control (*A*‒*H*). *I*, 293T cells were seeded in 96-well plates and cotransfected with MAVS and TRIM28-WT, TRIM28-ΔR or TRIM28-CA, along with NF-κB-luc or IFN-β-luc. A pRL-TK plasmid was cotransfected to normalize transfection efficiency. Data are presented as the mean ± SD and representative of three independent experiments. The statistical significance analyses were performed using two-sided unpaired *t* test. ∗∗*p* < 0.01; ∗∗∗*p* < 0.001; ∗∗∗∗*p* < 0.0001; and ns, not significant. HA, hemagglutinin; MAVS, mitochondrial antiviral signaling; TRIM28, tripartite motif 28.

K48-, K11- and K33-linked ubiquitination events are related to the regulation of proteasome-dependent degradation of substrate proteins and TRIM28, which has been previously reported to exhibit E3 ubiquitin ligase activity (21, 24, 25); we therefore sought to determine whether TRIM28 facilitates the K48-, K11-, or K33-linked ubiquitination of endogenous MAVS. The immunoblots showed that overexpression of TRIM28 enhanced the number of K48-linked polyubiquitin chains on endogenous MAVS (Fig. 5*A*). In contrast, TRIM28 exerted no effect on the K11- or K33-linked polyubiquitination of MAVS (Fig. 5, *B* and *C*). For confirmation, we applied K48R-Ub, a mutant that exclusively eliminates K48-linked polyubiquitination. Compared to K48-Ub, K48R ubiquitin markedly inhibited the TRIM28-mediated polyubiquitination of MAVS (Fig. 5*D*). Furthermore, we showed that the K48-linked polyubiquitination of exogenously expressed MAVS was enhanced by the overexpression of TRIM28 (Fig. 5*E*) but was decreased by the knockdown of TRIM28 (Fig. 5, *F* and *G*). These results demonstrate that TRIM28 mediates the K48-linked polyubiquitination of MAVS.

The C65 and C68 residues in the RING domain of TRIM28 have been previously identified to be critical for promoting ubiquitination (21). Thus, we constructed two TRIM28 mutants, TRIM28-ΔR (with the RING domain deleted) and TRIM28-CA (C65A/C68A), for further investigation. The results showed that in comparison with the TRIM28-WT construct, TRIM28-ΔR and TRIM28-CA nearly abolished the effect of TRIM28 on the K48-linked polyubiquitination of MAVS (Fig. 5*H*). Consistent with this finding, TRIM28-ΔR and TRIM28-CA completely abrogated the inhibitory effect of TRIM28 on MAVS-induced RLR signaling (Fig. 5*I*). Collectively, these data suggest that TRIM28 E3 ligase activity is critical for its suppressive effect on MAVS-dependent innate immune responses.

### The C-terminal domains of TRIM28 play key roles in binding to MAVS

**Figure 6:**
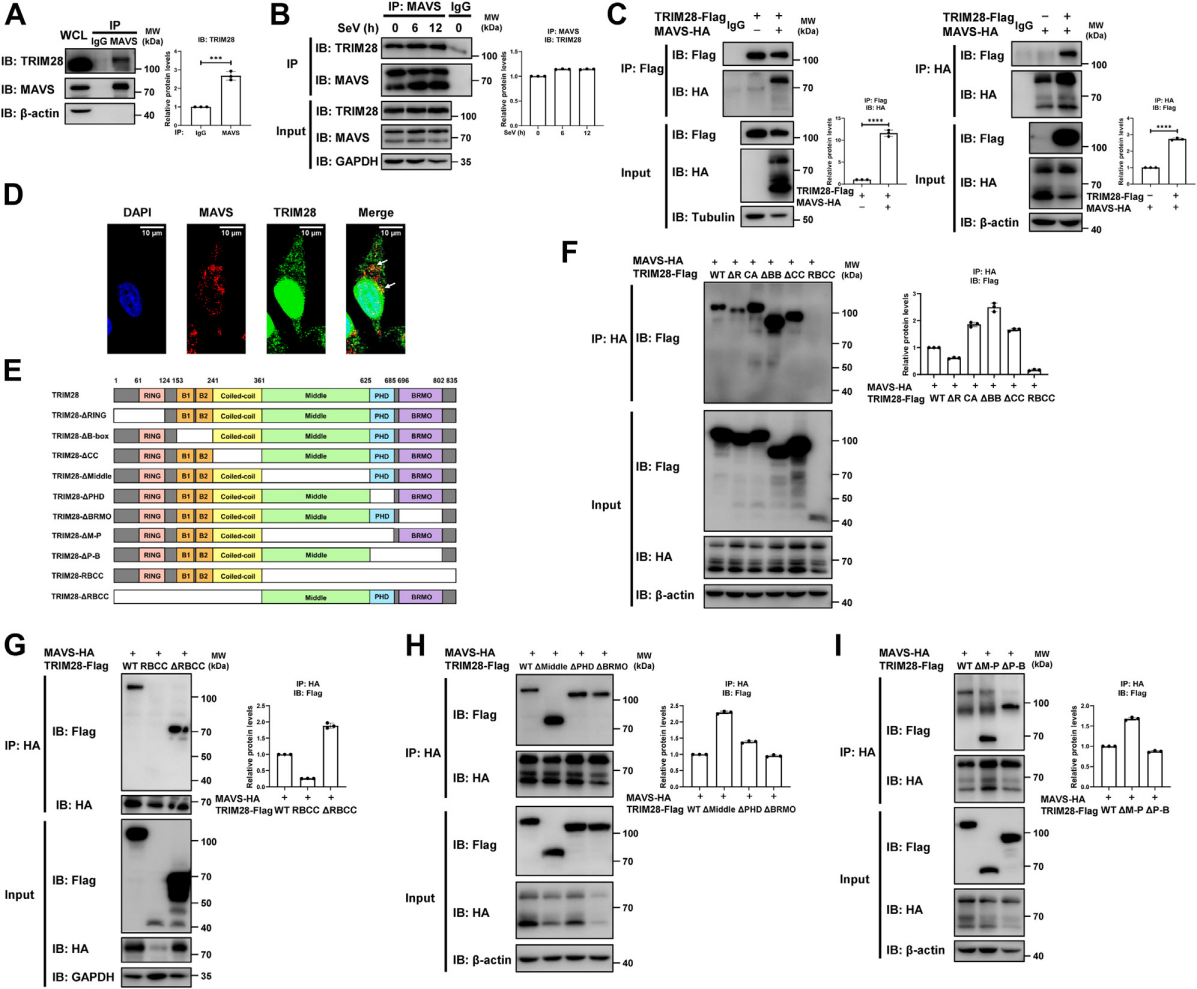
**The C-terminal domains of TRIM28 play key roles in binding to MAVS.***A*, coimmunoprecipitation (Co-IP) of 293T cells with an anti-MAVS antibody, followed by immunoblot analysis with an anti-TRIM28 antibody. *B*, 293T cells were infected with SeV (MOI = 1) for the indicated time periods. The cell lysates were immunoprecipitated with an anti-MAVS antibody and the interaction between endogenous TRIM28 and MAVS was analyzed by immunoblot analysis with an anti-TRIM28 antibody. *C*, Co-IP analysis of the interaction between ectopically expressed MAVS-HA and TRIM28-Flag in 293T cells. Immunoprecipitation and immunoblot analysis were performed with the indicated antibodies. *D*, immunofluorescence and confocal analysis of the localization of endogenous MAVS (*Red*) and TRIM28 (*Green*) in HeLa cells. *E*, schematic of TRIM28 mutants used in this study. *F*‒*I*, 293T cells were cotransfected with HA-tagged MAVS and Flag-tagged TRIM28 or the indicated mutants. The cell lysates were immunoprecipitated with an anti-HA antibody and then immunoblotted with an anti-Flag antibody. Protein bands (IP) were quantified and normalized to control (*A*‒*C*, and *F*‒*I*). Data are presented as the mean ± SD (n = 3). The statistical significance analyses were performed using two-sided unpaired *t* test. ∗∗∗*p* < 0.001; ∗∗∗∗*p* < 0.0001. HA, hemagglutinin; MAVS, mitochondrial antiviral signaling; MOI, multiplicity of infection; SeV, Sendai virus; TRIM28, tripartite motif 28.

Having identified that TRIM28 promotes K48-linked ubiquitination and the proteasomal degradation of MAVS, we next sought to determine whether TRIM28 directly bound to MAVS. Co-immunoprecipitation (Co-IP) assays revealed that endogenous TRIM28 interacted with endogenous MAVS (Fig. 6*A*). Moreover, the interaction between TRIM28 and MAVS was slightly enhanced in the presence of SeV infection (Fig. 6*B*). The association between exogenous Flag-tagged TRIM28 and HA-tagged MAVS was also examined and yielded similar results, providing further evidence for the presence of a TRIM28‒MAVS complex (Fig. 6*C*). In parallel, we analyzed the intracellular localization of endogenous TRIM28 and MAVS. Immunofluorescence experiments showed that TRIM28 colocalized with MAVS in HeLa cells (Fig. 6*D*).

TRIM28 is composed of an N-terminal tripartite RBCC motif, which contains a RING domain, two B-box domains and a coiled-coil domain, and C-terminal domains: a middle domain where the HP1-binding motif is located, plant-homeodomain (PHD), and bromodomain (26, 27, 28). To identify which domain(s) of TRIM28 is (are) required for its interaction with MAVS, we constructed a series of TRIM28 deletion mutants (Fig. 6*E*). First, we examined whether any domain in the RBCC motif is involved in the TRIM28‒MAVS interaction. The results showed that neither the ΔRING, ΔB-box, Δcoiled-coil mutants nor the TRIM28-CA mutant abolished the interaction with HA-tagged MAVS (Fig. 6*F*), suggesting that the N-terminal RBCC motif has no contribution to the formation of the TRIM28‒MAVS complex. Next, we investigated whether TRIM28-ΔRBCC, which lacks the entire RBCC domain, associates with MAVS. As shown in Figure 6*G*, the TRIM28-RBCC construct lost the ability to interact with MAVS as expected, while TRIM28-ΔRBCC bound to MAVS as potently as TRIM28-WT. These results indicate that the C-terminal domains are critical in mediating TRIM28 association with MAVS. Thus, we next constructed a series of TRIM28 mutants by deleting one or two of the C-terminal domains to identify the responsible domain(s). Co-IP analyses showed that each of the single domain–deletion mutants of TRIM28 retained a strong interaction with MAVS (Fig. 6*H*). Moreover, the mutant with both the middle and PHD domains deleted (TRIM28-ΔM-P) or with the PHD and bromodomain deleted (TRIM28-ΔP-B) bound to MAVS as strongly as TRIM28-WT (Fig. 6*I*). Thus, these results suggest that each of the C-terminal domains of TRIM28 plays a crucial role in the interplay between TRIM28 and MAVS.

### TRIM28 catalyzes K48-linked polyubiquitination of MAVS at multiple lysine residues

**Figure 7:**
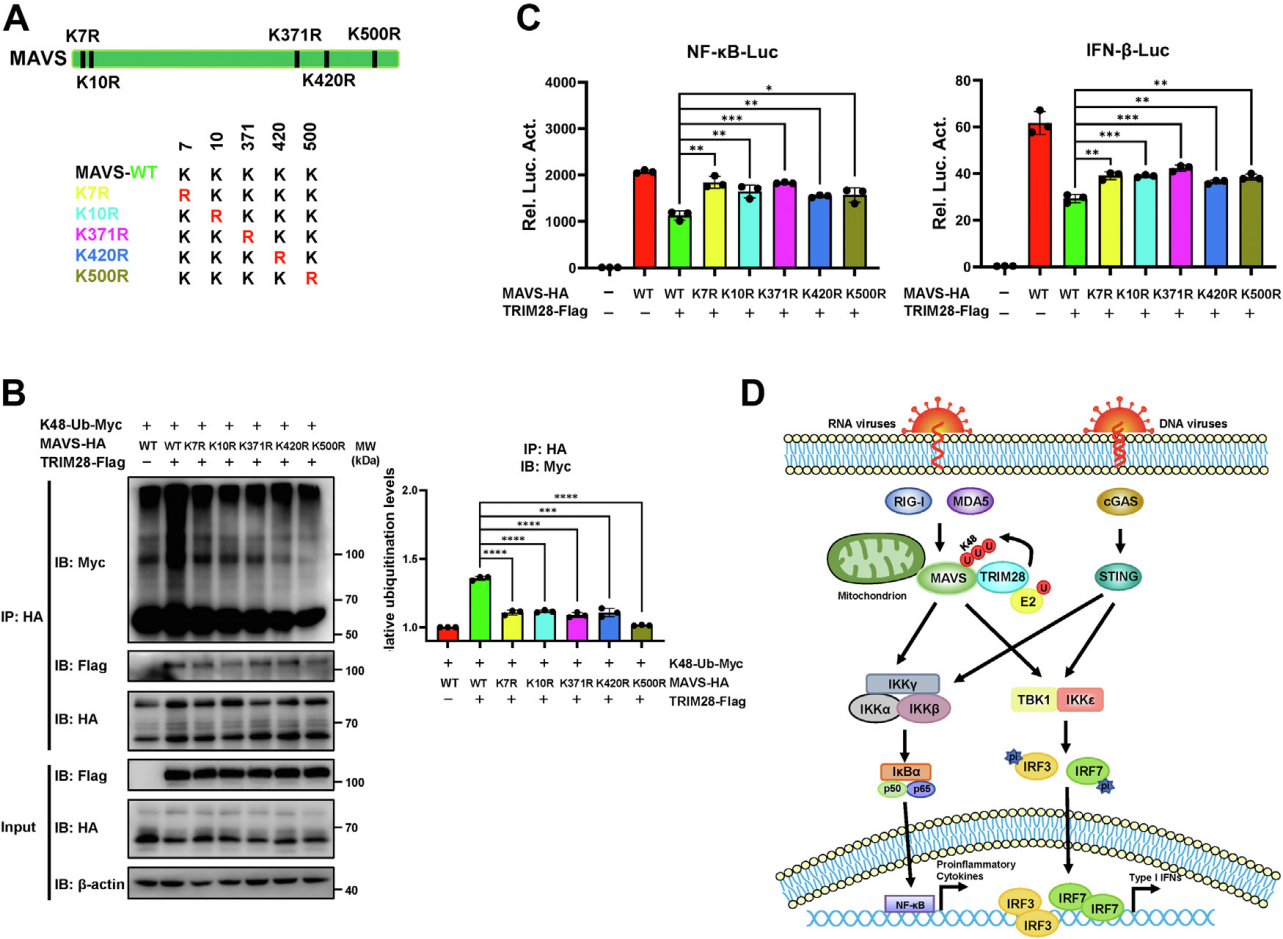
**TRIM28 catalyzes K48-linked polyubiquitination of MAVS at multiple lysine residues.***A*, schematic diagram showing point mutants of MAVS with lysine to arginine substitutions at residue 7, 10, 371, 420, or 500. *B*, ubiquitination assays of HA-tagged MAVS WT or mutants in 293T cells transfected with K48-Ub-Myc and TRIM28-Flag or an empty vector. The cell lysates were immunoprecipitated with an anti-HA antibody and the levels of ubiquitination were measured with an anti-Myc antibody. *C*, dual‒luciferase reporter assays of NF-κB-luc or IFN-β-luc in 293T cells transfected with TRIM28-Flag and HA-tagged MAVS WT or lysine mutants. A pRL-TK *Renilla* luciferase reporter construct was cotransfected as an internal control. All data are representative of three independent experiments and are presented as the mean ± SD (n = 3). The statistical significance analyses were performed using two-sided unpaired *t* test. ∗*p* < 0.05; ∗∗*p* < 0.01; ∗∗∗*p* < 0.001; ∗∗∗∗*p* < 0.0001. *D*, a proposed model for TRIM28-mediated inhibition of MAVS-dependent innate immune signaling. HA, hemagglutinin; IFN, interferon; MAVS, mitochondrial antiviral signaling; NF-κB, nuclear factor-κB; TRIM28, tripartite motif 28.

To gain further insight into the mechanisms by which TRIM28 degrades MAVS, we set out to identify the lysine residues of MAVS that are targeted by TRIM28. According to previous reports, several proteins have been demonstrated to mediate the degradation and K48-linked ubiquitination of MAVS, such as AIP4, Smurf1/Smurf2, RNF5, RNF125, MARCH5, and TRIM25 (12, 13, 14, 29, 30, 31, 32). These studies demonstrated that the ubiquitinated lysine (K) sites in MAVS include K7, K10, K371, K420, and K500. Thus, we mutated each of these lysine residues by replacing them with an arginine (R) (Fig. 7*A*). Co-IP analyses showed that all MAVS mutants exhibited a significant decrease in K48-linked polyubiquitination induced by TRIM28 in comparison with MAVS-WT (Fig. 7*B*). Consistently, dual‒luciferase reporter assays showed that the TRIM28-mediated suppression of MAVS-induced NF-κB-luc and IFN-β-luc was diminished when each of the tested lysine residues of MAVS was mutated (Fig. 7*C*). Taken together, these data demonstrate that TRIM28 catalyzes the K48-linked polyubiquitination of MAVS at the K7, K10, K371, K420, and K500 residues.

## Discussion

Timely and proper innate immune activation plays an important role in defending against pathogenic microorganisms, while persistent immune activation may lead to excessive inflammation and an imbalance in immune homeostasis. Therefore, it is of great importance to elucidate the mechanisms by which excessive immune reactions are prevented. Previously, TRIM28 was reported to inhibit the production of type Ⅰ IFNs and proinflammatory cytokines at the gene transcriptional level by targeting IRF7 and NF-κB/p65 (16, 18). In the present study, we report that TRIM28 directly suppresses the RLR signaling pathway by targeting MAVS for degradation *via* K48-linked polyubiquitination (Fig. 7*D*), providing evidence that TRIM28 functions as a dual regulator of antiviral innate immune responses.

Ubiquitination is crucial for the regulation of innate immune signaling pathways. Both K48-linked and K63-linked ubiquitin chains of key signaling molecules have been reported to participate in regulating antiviral immune responses (33). Increasing evidence has shown that TRIM family proteins are involved in modulating antiviral signaling and mediating ubiquitination of targeted proteins *via* their E3 ligase activities. For example, TRIM31 and TRIM35 positively regulate innate antiviral signaling by catalyzing the K63-linked polyubiquitination of MAVS and TRAF3, respectively (34, 35). TRIM29 inhibits the production of type Ⅰ IFNs by promoting MAVS degradation *via* K11-linked ubiquitin chains (24). TRIM25 conjugates K63-linked polyubiquitin chains on RIG-I and promotes the K48-linked ubiquitination and proteasomal degradation of MAVS (11, 14), revealing that TRIM25 plays a crucial dual role in the RLR signaling pathway. Previous studies have shown that TRIM28 negatively regulates RLR signaling by targeting transcription factors such as IRF7 and NF-κB/p65 (16, 18). In this study, we provide evidence to support TRIM28 as a negative regulator of the RLR signaling pathway, with a previously uncharacterized MAVS-dependent mechanism.

In the RLR innate immune signaling pathway, MAVS is a key adapter that recruits downstream signaling factors, ultimately leading to the activation of IFNs and the production of proinflammatory cytokines. The suppression of MAVS blocks IFN production and increases viral replication (7). Several E3 ubiquitin ligases have been shown to mediate the ubiquitination of MAVS and thus participate in regulating antiviral signaling; these ligases include HECT domain-containing E3 ligase AIP4 (12), Smurf1/2 (29, 30), and RING-type E3 ligase RNF5 (31), RNF125 (13), MARCH5 (32), TRIM21 (36), TRIM25 (14), TRIM29 (24), and TRIM31 (34). Different preferences of the ubiquitinated lysine sites of MAVS targeted by those ligases were demonstrated. For example, AIP4 mediates MAVS ubiquitination at K371 and K420 (12), TRIM25 targets MAVS at K7 and K10 for K48-linked ubiquitination (14), and MARCH5 transfers ubiquitin to K7 and K500 of MAVS *via* a K48 linkage (32). In our study, we demonstrate that the K7, K10, K371, K420, and K500 residues of MAVS are targets for K48-linked polyubiquitination by TRIM28, providing new insights into the mechanisms underlying the ubiquitination of MAVS.

TRIM28 was initially identified as a crucial transcriptional corepressor for Kruppel-associated box-zinc finger proteins (37, 38, 39) and was believed to act as a scaffold for heterochromatin protein HP1, histone methyltransferase SETDB1, and histone deacetylase-containing complex NuRD for the formation of heterochromatin (40, 41, 42). With accumulating evidence, the importance of TRIM28 in regulating innate immune responses has started to emerge. Tsuruma *et al.* (17) reported that a reduction in TRIM28 expression enhanced IL-6–induced STAT3-dependent gene expression by dephosphorylating STAT3. Kamitani *et al.* (18) reported that TRIM28 was involved in the deacetylation of NF-κB/p65 by interfering with the interactions among STAT3, p300, and NF-κB/p65, which led to reduced TNF-α-activated IL-6 production. Liang *et al.* (16) demonstrated that TRIM28 inhibits IRF7 transactivation activity by SUMOylating IRF7, leading to compromised IFN production and antiviral responses. Notably, all of these studies indicate that TRIM28 negatively regulates the innate immune response at the transcriptional level of immune-related genes. In our study, we determined that TRIM28 directly interferes with the RIG-I‒MAVS but not the STING signaling pathway axis by degrading the key signal transducer MAVS, resulting in an inhibitory effect on RNA virus–triggered IFN production and proinflammatory cytokines. Thus, our findings may partly explain why TRIM28 abolished the suppressive effect on IFN-β promoter activity in MAVS-KO cells but not in STING-KO cells, which was reported by Tie *et al.* (19).

In summary, we identified the E3 ubiquitin ligase TRIM28 as a negative regulator of MAVS-dependent IFN responses. TRIM28 interacts with MAVS through its C-terminal domains and promotes the K48-linked ubiquitination of MAVS at multiple lysine residues. Our findings uncover a previously uncharacterized role for TRIM28 in fine-tuning innate immune responses and provide novel insights into the molecular mechanisms by which MAVS-mediated RLR signaling is negatively regulated, contributing to our understanding of the cellular homeostatic control of innate immunity.

## Experimental procedures

### Cell culture

Human embryonic kidney 293 T cells and HeLa cells were maintained in Dulbecco’s modified Eagle’s medium supplemented with 10% certified fetal bovine serum (FBS; VivaCell). All cell lines have been tested negative for *mycoplasma* contamination using a PCR-based *mycoplasma* detection kit (C0301S, Beyotime Biotec) and cultured at 37 °C in a 5% CO_2_ incubator.

### Antibodies and reagents

The following antibodies were used in this study: rabbit anti-KAP1 (AF2620, Beyotime Biotec), rabbit anti-HA (H6908, Sigma-Aldrich). Rabbit anti-MAVS (14341-1), rabbit anti-RIG-I (20566-1), rabbit anti-Myc (16286-1), rabbit anti-Flag (20543-1), mouse anti-TRAF6 (66498-1), mouse anti-GAPDH (60004-1), mouse anti-Tubulin (66031-1), goat anti-mouse IgG-HRP, and goat anti-rabbit IgG-HRP were from Proteintech. Mouse anti-MAVS (sc-166583) and mouse anti-Flag (sc-7392) were from Santa Cruz Biotechnology. Mouse anti-HA (201113), mouse anti-β-Actin (200068-8F10), and rabbit anti-IRF3 (381333) antibodies were purchased from Zen-Bioscience. Rabbit anti-HA (3724S), anti-TBK1 (3504S), anti-p-TBK1 (5483SS), anti-p-IRF3 (29047S), anti-p-IκBα (2859S), and mouse anti-IκBα (4814S) were purchased from Cell Signaling Technology.

SeV was obtained as previously described and the multiplicity of infection was 1. Proteasome inhibitor MG132 (S1748, Beyotime Biotec) and (S7418, Selleck) were dissolved in dimethyl sulfoxide (D8418, Sigma-Aldrich). The autolysosome inhibitor bafilomycin A1 (BafA1; 88899-55-2, Aladdin) was kindly provided by Prof. Rui Cheng (Chongqing Medical University). MG132 (10 μM) and BafA1 (5 nM) were used for 6 h, and CHX was used at 100 μg/ml for indicated time.

### Plasmids construction and virus production

The plasmids used in the study were constructed as follows. TRIM28 core domain were obtained from the HEK293T cDNA library, tagged with a Flag peptide, and cloned into the eukaryotic expression vector pLPCX. Domain-deletion mutants of TRIM28 were generated by overlapping PCR. The primers were listed in Table S1.

The pLPCX vectors encoding HA-tagged N-terminal RIG-I, MAVS and STING were generated by PCR amplification in human cDNA with the primers listed in Table S1. HA-tagged deletion constructs of MAVS were generated by the point-mutation method. The primers used were listed in Table S1. pGL4.32-NF-κB-luc, pGL4.32-IFN-β-luc, pRL-TK (*Renilla* luciferase), and Myc-tagged wildtype ubiquitin (WT-Ub-Myc) were kindly provided by Dr Yong-Tang Zheng (Kunming Institute of Zoology, Chinese Academy of Sciences). K48-Ub-Myc and K48R-Ub-Myc were synthesized and then cloned into WT-Ub-Myc with HindⅢ and SalI. Myc-tagged K11 Ubiquitin (K11-Ub-Myc) and Myc-tagged K33 Ubiquitin (K33-Ub-Myc) were kindly provided by Prof. Zan Shen (Chongqing Medical University).

shRNA target sequences cloned into pLKO.1 were as follows: scramble: 5′-CCTAAGGTTAAGTCGCCCTCG-3′; TRIM28: 5′-GCAACAGTGCTTCTCCAAAGA-3′; MAVS: 5′-CAAGTTGCCAACTAGCTCAAA-3′. VSV-G-pseudotyped lentiviral vectors were produced by cotransfecting HEK293T cells using polyethylenimine (24765-1, Polysciences) in 6-well plates with the shRNA plasmid, psPAX2 and pMD2.G encoding VSV-G at 4:3:1 ratio. The supernatant was harvested 48 h after transfection.

### Generation of stably transduced cell lines

HEK293T cells were seeded into 6-well plates at a density of 2 × 10^5^ cells/ml and were transduced with shRNA-encoding lentivirus. Forty-eight hours postincubation, the cells were reseeded into 6-well plates with fresh Dulbecco's modified Eagle's medium with 10% FBS and were selected by puromycin (1 μg/ml; ant-pr-5, Invivogen). After 48 h selection, immunoblot analysis was performed to determine the knockdown efficiency.

### Dual‒luciferase assay

Luciferase assays were conducted according to the Promega Dual Luciferase Kit instructions. HEK293T cells were seeded in 96-well plates overnight and then cotransfected with an NF-κB luciferase reporter (100 ng) or an IFN-β luciferase reporter (100 ng) together with pRL-TK plasmid expressing *Renilla* luciferase (20 ng), as well as different concentration of MAVS-HA, TRIM28-Flag, or empty vector pLPCX (50/100/200 ng) using PEI or Lipofectamine 2000 (Invitrogen, 11668-019) as described by the manufacturer’s instructions. SeV (multiplicity of infection = 1) was used as for activation for 24 h in the assay. Forty-eight hours posttransfection, the cells were collected, and luciferase activity was measured. Each experiment was carried out in triplicates. For each sample, firefly luciferase fluorescence units were normalized to *Renilla* luciferase fluorescence units to obtain relative fluorescence units.

### Co-immunoprecipitation and Western blot analysis

To exact the whole-cell lysates, cells were harvested and washed twice with PBS and then lysed in the denaturing radioimmunoprecipitation assay lysis buffer (1% NP40, 150 mM NaCl, 50 mM Tris-HCl [pH 8.0], 0.1% SDS, 0.5% sodium deoxycholate) supplemented with 1 mM phenylmethylsulfonylfluoride (ST506, Beyotime Biotec) and protease inhibitor cocktail (78425, Thermo Fisher Scientific) for 30 min on ice. The lysates were clarified by centrifugation at 12,000 rpm for 10 min at 4 °C. Cell lysates were separated by sodium dodecyl sulfate-polyacrylamide gel electrophoresis (SDS-PAGE) and then transferred to polyvinylidene difluoride membranes (Millipore). The blots were probed with indicated primary antibodies, followed by an IgG-peroxidase–conjugated secondary antibody. Immunoblots were visualized using the immobilon western chemiluminescent HRP substrate (Millipore). Grayscale values were measured using ImageJ software.

For Co-IP analyses, different tagged protein-expression constructs were transfected into HEK293T cells, which were cultured in 6 cm dishes, using PEI. Forty-eight hours posttransfection, cells were lysed in radioimmunoprecipitation assay lysis buffer containing 1 mM phenylmethylsulfonylfluoride and protease inhibitor cocktail for 30 min on ice. Cell lysates were centrifuged at 12,000 rpm for 10 min at 4 °C, and 400 μl of the clarified supernatant was used for immunoprecipitation; the remaining 50 μl lysate was diluted in 5× SDS-PAGE loading buffer, incubated at 100 °C for 5 min. Rabbit anti-HA, Mouse anti-MAVS, Mouse anti-Flag or control IgG (2 μg; Santa Cruz Biotechnology) was conjugated to 25 μl of Protein A/G magnetic beads (HY-K0202, MedChemExpress) and incubated for 2 h at 4 °C. The beads were washed four times with PBST buffer (1× phosphate-buffered saline [PBS] containing 0.5% Tween-20, pH 7.4), added to 400 μl cell extract, and then incubated for another 2 h at 4 °C. After washing, the pellet was resuspended in 60 μl 1× SDS-PAGE loading buffer, and the mixture was boiled at 100 °C for 5 min. Samples were separated by SDS-PAGE and immunoblotted with indicated primary and secondary antibodies.

### RNA isolation, reverse transcription, and real-time quantitative PCR

Total RNA was extracted from HEK293T cells dissolved in TRIzol reagent (TaKaRa) and reverse transcribed into cDNA using the RevertAid First Strand cDNA Synthesis Kit (Thermo Fisher Scientific). Real-time quantitative PCR was performed on a Bio-Rad CFX96 real-time PCR system. The following primers were used: *TRIM28* forward, 5′-GCAGAGCGTCCTGGCACTAAC-3′ and reverse, 5′-CCTGACCCAAAGCCATAGCCT-3′; *IFNB1* forward, 5′-CCAACAAGTGTCTCCTCCAAAT-3′ and reverse, 5′-AATCTCCTCAGGGATGTCAAAG-3′; *CXCL10* forward, 5′-TCCACGTGTTGAGATCATTGC-3′ and reverse, 5′-TCTTGATGGCCTTCGATTCTG-3′; *ISG56* forward, 5′-CCTGAAAGGCCAGAATGAGG-3′ and reverse, 5′-TCCACCTTGTCCAGGTAAGT-3′; *MAVS* forward, 5′-GTGCCTACTAGCATGGTGCTC-3′ and reverse, 5′-GACCCAAGGCCCCTATTCT-3′; *GAPDH* forward, 5′-GCTTCGCTCTCTGCTCCTCCTGTT-3′ and reverse 5′-ACGACCAAATCCGTTGACTCCGACC-3′.

### Cell viability assay

Cell viability was examined using a Cell Counting Kit-8 (CCK-8; B34304, Bimake) assay. Cells were cultured in a 96-well plate at concentration of 5 × 10^4^ cells/ml, and *A*450 was measured 1.5 h after adding CCK-8 at 0, 24, 48, and 72 h with a microplate reader.

### Immunofluorescence and confocal microscopy

HeLa cells were seeded on coverslips in 24-well plates overnight. Twenty-four hours posttransfection, cells were washed with PBS, fixed with 4% paraformaldehyde for 10 min, washed with PBS three times, permeabilized with 0.1% Triton X-100 for 10 min. After washing with PBS three times, cells were blocked for 1 h at room temperature and incubated with appropriate primary antibodies overnight at 4 °C. Secondary antibodies were applied for 2 h at room temperature. Cell nuclei were stained with DAPI (S2110, Solarbio). Confocal immunofluorescence microscopy was carried out using an Andor2000 microscope (Oxford instrument, UK). Obtained images were processed and analyzed with Fiji software.

### Statistical analysis

Data were presented as means ± SD values. The statistical significance analyses were performed by two-sided unpaired *t* test (*p*-values) using Prism 8 software (GraphPad). Differences were considered significant at a *p*-value < 0.05. The levels of significance were indicated as follows: ∗*p* < 0.05, ∗∗*p* < 0.01, ∗∗∗*p* < 0.001, ∗∗∗∗*p* < 0.0001, and ns indicated no significance.

## Data availability

All data supporting this study have been included within the article and supporting files.

## Supporting information

This article contains supporting information.

## Conflict of interest

The authors declare that they have no conflicts of interest with the contents of this article.
