# Supplementary material for: TRIM28 negatively regulates the RLR signaling pathway by targeting MAVS for degradation via K48-linked polyubiquitination
Source: J Biol Chem. 2023 Apr 27;299(5):104660. doi: 10.1016/j.jbc.2023.104660 (PMC10165269; doi:10.1016/j.jbc.2023.104660)
Supplement: Supporting Table S1 [file mmc1.docx]

Table S1. Primers used in the study.

| Primers | Sequences (5’ - 3’) |
| --- | --- |
| pLPCX-TRIM28-Flag  Forward primer | CCGCTCGAGGCCACCATGGCGGCCTCCGCG |
| pLPCX- TRIM28-Flag  Reverse primer | TATCCATCGATTCACTTATCGTCGTCATCCTTGTAATCGCCGCCGGGGCCATCACCAGGGCCACCA |
| pLPCX-N-RIG-I-HA  Forward primer | CCGCTCGAGGCCACCATGACCACCGAGCAGCGACG |
| pLPCX-N-RIG-I-HA  Reverse primer | CATCTTAAAAAAGGCGGCTACCCATACGACGTCCCA GACTACGCTTGAATCGATGGATA |
| pLPCX-STING  Forward primer | CTCGAGGCCACCATGCCCCACTCCAGCCTGCATC |
| pLPCX-STING  Reverse primer | TCAAGAGAAATCCGTGCGGAGAG |
| pLPCX-MAVS-HA  Forward primer | CCGCTCGAGGCCACCATGCCGTTTGCTGAAGACAAGACCTATAAG |
| pLPCX-MAVS-HA  Reverse primer | CCATCGATTCAAGCGTAGTCTGGGACGTCGTATGGGTAGCCGCCGTGCAGACGCCGCC |
| pLPCX-TRIM28-ΔR-Flag  Forward primer | GCGCCGAGGCGCTGGAGTTCTCCAAAGACATCGTGG |
| pLPCX-TRIM28-ΔR-Flag  Reverse primer | CCACGATGTCTTTGGAGAACTCCAGCGCCTCGGCGC |
| pLPCX-TRIM28-C65AC68A-Flag  Forward primer | GAGCACGCCGGCGTGGCCAGAGAGCGCCTGCGAC |
| pLPCX-TRIM28-C65AC68A-Flag  Reverse primer | CGCAGGCGCTCTCTGGCCACGCCGGCGTGCTCCAGCAGCTCCAGCG |
| pLPCX-TRIM28-ΔBB-Flag  Forward primer | CAGGATGCGAACCAGTGCTACCAGTTCTTAGAGG |
| pLPCX-TRIM28-ΔBB-Flag  Reverse primer | CCTCTAAGAACTGGTAGCACTGGTTCGCATCCTG |
| pLPCX-TRIM28-ΔCC-Flag  Forward primer | CCACAAGGACCACCAGTTGCTTTCTAAGAAGTTG |
| pLPCX-TRIM28-ΔCC-Flag  Reverse primer | CAACTTCTTAGAAAGCAACTGGTGGTCCTTGTGG |
| pLPCX-TRIM28-RBCC-Flag  Reverse primer | TTGCGGCCGCTCATCACTTATCGTCGTCATCCTTGTAATCGCCGCCGCACTGTTGCTTG |
| pLPCX-TRIM28-ΔRBCC-Flag  Forward primer | CGGAATTCGCCACCATGTTGCTTTCTAAGAAG |
| pLPCX-TRIM28-ΔMiddle-Flag  Forward primer | CAACAACACAGCCCTTGCCACCATTTGCCGTG |
| pLPCX-TRIM28-ΔMiddle-Flag  Reverse primer | CACGGCAAATGGTGGCAAGGGCTGTGTTGTTG |
| pLPCX-TRIM28-ΔPHD-Flag  Forward primer | GAACCCTGGATGACAGTGGTGCAGACAGCACTG |
| pLPCX-TRIM28-ΔPHD-Flag  Reverse primer | CAGTGCTGTCTGCACCACTGTCATCCAGGGTTC |
| pLPCX-TRIM28-ΔBRMO-Flag  Forward primer | GCGTGGTGGCCAAGACCAAGTTCTCTGCTG |
| pLPCX-TRIM28-ΔBRMO-Flag  Reverse primer | CAGCAGAGAACTTGGTCTTGGCCACCACGC |
| pLPCX-MAVS-K7R-HA  Forward primer | CCGCTCGAGGCCACCATGCCGTTTGCTGAAGACAGGACCTATAAGTATATC |
| pLPCX-MAVS-K10R-HA  Forward primer | CCGCTCGAGGCCACCATGCCGTTTGCTGAAGACAAGACCTATAGGTATATCTGCCGCAAT |
| pLPCX-MAVS-K371R-HA  Forward primer | CTAGCATGGTGCTCACCAGGGTGTCTGCCAGCACAG |
| pLPCX-MAVS-K371R-HA  Reverse primer | CTGTGCTGGCAGACACCCTGGTGAGCACCATGCTAG |
| pLPCX-MAVS-K420R-HA  Forward primer | GGTCGGAGCTGAGTAGACCTGGCGTGCTGGC |
| pLPCX-MAVS-K420R-HA  Reverse primer | GCCAGCACGCCAGGTCTACTCAGCTCCGACC |
| pLPCX-MAVS-K500R-HA  Forward primer | CACAAGCCGACCGGAGGTTCCAGGAGAGGGAG |
| pLPCX-MAVS-K500R-HA  Reverse primer | CTCCCTCTCCTGGAACCTCCGGTCGGCTTGTG |
| HindⅢ-WT-Ub-Myc  Forward primer | CCAAGCTTATTTCTGAAGAGGACTTGG |
| SalI-WT-Ub  Reverse primer | GCGTCGACTCCTTCTGAATGTTGTAATC |
| K48R-Ub-Myc  Forward primer | TGATCTTTGCCGGTAGGCAGCTCGAGGACGGTAG |
| K48R-Ub-Myc  Reverse primer | CCTCGAGCTGCCTACCGGCAAAGATCAATCTTTG |
